# Supplementary figures and images for: The Chalcone Isomerase Family in Cotton: Whole-Genome Bioinformatic and Expression Analyses of the Gossypium barbadense L. Response to Fusarium Wilt Infection
Source: Genes (Basel). 2019 Dec 4;10(12):1006. doi: 10.3390/genes10121006 (PMC6947653; doi:10.3390/genes10121006)

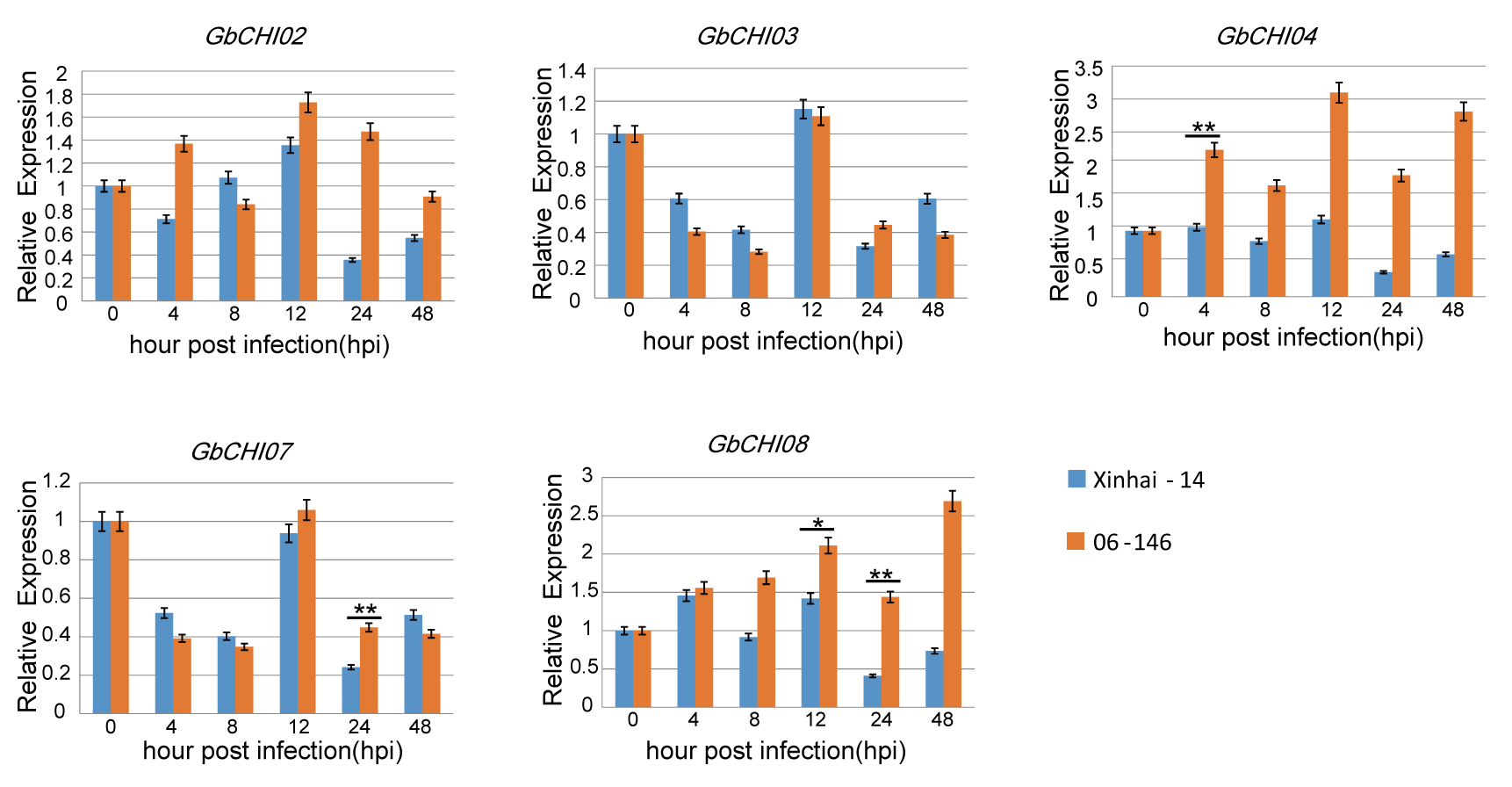

Supplement: Supplementary file 1 [file genes-10-01006-s001.zip › Supplementary File(s)/Figure S1.tif]
